# Malignant melanoma with pituitary metastasis: A case report and literature review

**DOI:** 10.3389/fendo.2025.1661983

**Published:** 2025-10-06

**Authors:** Xiaoling Li, Wenhui Jiang, Xiaohui Tang, Meiying Chen, Wenhua Deng, Yunyu Wang, Xingfu Wang

**Affiliations:** ^1^ Department of Pathology, The Second Hospital of Longyan, Longyan, China; ^2^ Molecular Biology Laboratory, The Second Hospital of Longyan, Longyan, China; ^3^ Department of Pathology, The First Affiliated Hospital of Fujian Medical University, Fuzhou, China

**Keywords:** pituitary metastasis, malignant melanoma, multimodal diagnosis, *BRAF* V600E, case report

## Abstract

**Purpose:**

Pituitary metastasis of malignant melanoma (MM) is rare. This study aimed to explore its diagnostic features using a multimodal approach and retrospectively analyzed previously reported cases to summarize its pathogenesis and diagnostic challenges.

**Methods:**

We screened all published case reports and case series on pituitary metastatic MM using PubMed, focusing on cases with detailed clinical data, imaging features, pathological examination, and molecular test results. A total of 24 cases of MM with pituitary metastasis, including our case, were retrospectively analyzed. Additionally, the index patient underwent histopathological, immunohistochemical (S100, SOX10, Melan-A, HMB-45, *BRAF* V600E), and *BRAF* V600E PCR analyses.

**Results:**

This case involved a 65-year-old female patient whose pathological examination revealed tumor cells with epithelioid and spindle cell features. Immunohistochemical analysis showed diffuse positivity for S-100, vimentin, and *BRAF* V600E, with focal positivity for Melan-A and HMB-45. The Ki-67 proliferation index was approximately 15%. Molecular testing confirmed *BRAF* V600E mutation. The patient died 12 months postoperatively. Our literature review indicated that MM with pituitary metastasis demonstrates male predominance, a median onset age of 62 years, a frequent association with *BRAF* V600E mutation, and a median survival time of 12 months.

**Conclusion:**

Diagnosing MM with pituitary metastasis requires integrating detailed clinical history, imaging features, pathological examination, and molecular testing. Our findings highlight the importance of a comprehensive diagnostic approach with multidisciplinary collaboration when managing atypical pituitary masses, along with detailed investigation of a patient’s previous tumor history, to improve diagnostic accuracy and patient outcomes.

## Introduction

The sellar region of the central nervous system is anatomically and functionally critical owing to its proximity to numerous vital structures. Pituitary adenomas are the most common tumors in this area, followed by craniopharyngiomas. Less common tumors include granulosa cell tumors, pituitary cell tumors, spindle cell eosinophilic tumors, and, rarely, pituitary metastases. Pituitary metastases account for only 1%–4% of all pituitary tumors (1, 2), with breast and lung cancers being the most common primary tumors (3, 4). Renal and prostate cancers are other frequent sources of metastasis (5), and virtually any type of tumor can metastasize to the pituitary region (6), including malignant melanoma (MM) (7, 8). Cutaneous melanoma most frequently metastasizes to lung, liver, brain, and bone. Approximately 50% of advanced melanomas harbour *BRAF* V600E mutations, making *BRAF* V600E analysis crucial for both diagnosis and targeted therapy. Pituitary metastasis of MM is particularly rare, and its imaging features often overlap with those of pituitary adenomas, making preoperative diagnosis extremely challenging.

The clinical presentation of pituitary metastasis is often nonspecific, with approximately 20% of patients presenting with symptoms that typically emerge in the advanced stages of the disease (3). Common manifestations include headache, visual impairment, cranial neuropathy, and pituitary dysfunction (3). These nonspecific symptoms pose significant diagnostic challenges, particularly when the primary tumor is unidentified. In this context, a comprehensive diagnostic approach is essential for accurately identifying and managing these rare cases.

In recent years, continuous advancements in imaging, pathological examination, and molecular diagnostic techniques have led to the gradual adoption of a multimodal diagnostic system for identifying nervous system tumors (9). Integrating detailed clinical history, imaging findings, pathological features, and molecular detection results can significantly improve the diagnostic accuracy of rare metastases. Additionally, targeted therapies against the *BRAF* V600E mutation have yielded remarkable progress in MM treatment. A landmark study demonstrated that patients with metastatic MM harboring the *BRAF* V600E mutation treated with dabrafenib–trametinib achieved a 5-year survival rate of 28% and an overall response rate of 76%, with 17% of the patients achieving complete remission (10). Long-term complete remission has also been reported, even after the treatment had been discontinued for 18 months (11). These findings indicate the significance of detecting the presence of a *BRAF* V600 mutation in MM with pituitary metastasis.

In this study, we report a case of MM with pituitary metastasis, discuss its clinical and imaging features, and highlight its diagnostic challenges. Combined with a literature review, we emphasize the value of a multimodal diagnostic system in accurately identifying rare pituitary metastases. Through this case study, we aim to provide clinicians and pathologists with a reference for diagnosing and treating such rare cases.

## Case report

**Figure 1:**
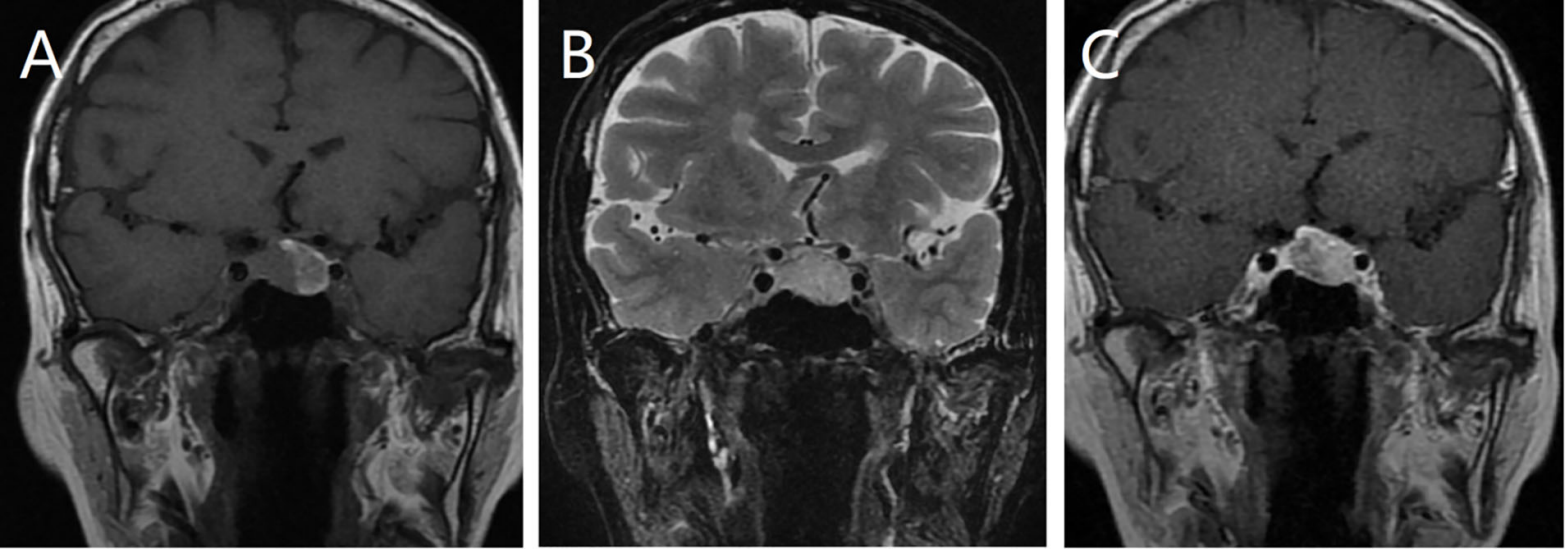
Magnetic resonance imaging (MRI) scans showing a circular abnormal signal shadow on the left side of the sellar area. The lesion exhibits an equal to slightly higher signal on T1-weighted imaging (T1WI) **(A)** and a slightly higher signal on T2-weighted imaging (T2WI) **(B)**, with clear boundaries. Contrast-enhanced MRI showing progressive inhomogeneous enhancement **(C)**.

A 65-year-old female patient presented with a 5-month history of dizziness. Hormonal evaluation revealed abnormal cortisol levels of 66.93 nmol/L, 130.50 nmol/L, and 124.84 nmol/L at 0, 8, and 16 h, respectively, as measured using the electrochemiluminescence immunoassay method (reference range: 171.0–536.0 nmol/L). All other hormone levels were within the normal limits. Cranial magnetic resonance imaging (MRI) (**Figure 1**) showed a round, abnormal signal shadow in the left sellar region. T1-weighted imaging (T1WI) revealed isointense to slightly hyperintense signals, while T2-weighted imaging (T2WI) showed slightly hyperintense signals. The lesion had clear boundaries and measured approximately 1.7 × 1.5 cm (coronal measurement). Contrast-enhanced scans revealed progressive, uneven, and marked enhancement without evidence of sellar floor bone absorption or destruction. A preliminary diagnosis of pituitary macroadenoma was made.

**Figure 2:**
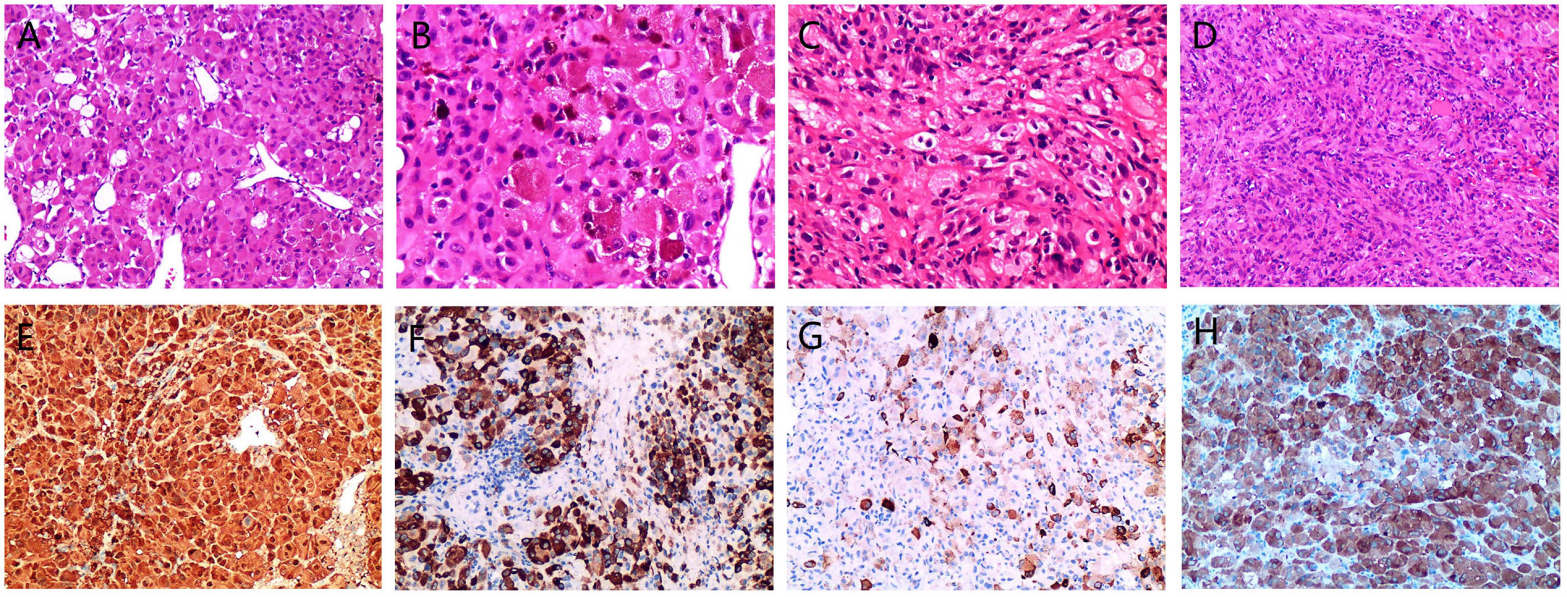
Microscopic morphology and immunohistochemical expression of pituitary and axillary metastatic malignant melanoma (MM). Tumor cells arranged in sheets with epithelioid morphology and thin-walled blood vessels are visible, HE, ×200 **(A)**. Epithelioid, polygonal cells displaying varying degrees of eosinophilia, with some cells containing pigment in the cytoplasm, HE, ×400 **(B)**. Spindle-shaped cells interwoven with epithelioid cells, HE, ×400 **(C)**. The axillary mass is predominantly composed of spindle-shaped cells, HE, ×200 **(D)**. Strongly positive for S-100, EnVision, ×200 **(E)**. Melan-A is strongly positive in most areas, EnVision,×200 **(F)**. HMB-45 exhibits varying degrees of expression in scattered cells, EnVision, ×200 **(G)**. Strongly positive for *BRAF* V600E,EnVision, ×200 **(H)**.

Intraoperative findings identified a solid mass in the sellar region with friable, fish-like tissue. Pathological examination revealed gray-brown, fragmented tumor tissue measuring 3 × 3 × 0.3 cm that was soft in texture. Microscopic evaluation revealed diffuse and patchy tumor cells, predominantly polygonal epithelioid cells with an eosinophilic cytoplasm (**Figure 2A**). Some cells exhibited a foamy cytoplasm with pigment deposition (**Figure 2B**). The nuclei were pleomorphic, including round, oval, and irregular shapes, with nucleoli visible in some cells (**Figure 2B**). A small number of spindle-shaped cells were interwoven with epithelioid cells, with deeply stained nuclei and inconspicuous nucleoli (**Figure 2C**). Mitotic figures were observed in both epithelioid and spindle cells, and focal lymphocytic aggregation and necrotic areas were present in the stroma. Immunohistochemical analysis showed diffuse positivity for S-100 and vimentin, with strong galectin-3 positivity. TTF-1, broad-spectrum CK, EMA, CgA, Syn, and GFAP were not expressed. The Ki-67 proliferation index was approximately 15%.

Prior to surgery, the patient reported a palpable axillary mass. Ultrasonography revealed a hypoechoic nodule in the right axilla. Intraoperatively, a mass approximately 3 cm in size was identified under the skin of the right axilla, with a smooth, cystic-solid appearance and containing a dark red fluid. The solid area had a fish-like texture with clear boundaries from the surrounding adipose tissue. Microscopic cell morphology and immunohistochemical expression of the axillary mass were similar to those of the sellar mass: predominantly spindle cells with few mitotic figures and no apparent pigment deposition (**Figure 2D**).

Given the simultaneous presence of an axillary mass, metastasis was suspected. The presence of pigment in the cytoplasm of the pituitary tumor cells and immunohistochemical expression of S-100 and vimentin were consistent with the characteristics of MM. Further review of the patient’s medical history revealed surgical excision of a nevus from the calf over 10 years ago, with difficult postoperative wound healing. This information supported the hypothesis that both the pituitary and axillary masses were metastatic MM. To confirm this diagnosis, immunohistochemical tests for Melan-A (**Figure 2F**), HMB45 (**Figure 2G**), and *BRAF* V600E (**Figure 2H**) were performed, all of which showed strong positivity, thereby supporting the diagnosis of MM.

*BRAF* V600E mutational status was determined by allele-specific real-time PCR (TaqMan^®^ SNP Genotyping Assay, ThermoFisher, sensitivity ≥1% mutant allele), validated in-house with known positive and negative controls, and detected the V600E mutation in both pituitary and axillary specimens. Ten days after surgery, the patient received dacarbazine plus ifosfamide; *BRAF*/MEK inhibitors were not used because the *BRAF* V600E status had not yet been determined. After two treatment cycles, the patient developed severe bone marrow suppression, discontinued therapy, and ultimately succumbed 12 months post-operatively.

## Literature review

To better understand the clinical manifestations and diagnostic approaches for MM with pituitary metastasis, a comprehensive PubMed search was conducted without date restrictions (up to 30 April 2024) using the Boolean query (“melanoma” OR “melanoma metastasis”) AND (“pituitary” OR “sella” OR “brain”). After excluding duplicates, primary melanoma, and non-metastatic lesions, 24 histologically confirmed cases of pituitary metastatic melanoma were retained for retrospective analysis (5, 7, 11–28) (**Table 1**). Although we attempted to identify all relevant cases, the possibility of publication or database bias could not be excluded. The cohort comprised 16 male patients, 7 female patients, and 1 patient of unknown sex (22). The patients’ ages ranged from 25 to 78 years, with a median age of 62 years. The primary clinical manifestations included visual impairment and pituitary dysfunction (3, 29), followed by headache and dizziness.

**Table 1:** Cases of malignant melanoma with pituitary metastasis described in the literature.

No.	Author/Year	Sex/Age (years)	Symptoms	Primary site/stage	MRI findings	Treatment	Time to metastasis (months)	Prognosis	*BRAF* V600E
1	Mayr et al. /1993 (16)	M/25	Pituitary dysfunction, optic nerve involvement	NA	T1WI high signal, T2WI isointense, enhancement visible	NA	25	NA	NA
2	Leung et al. /2003 (17)	M/46	Diabetes insipidus, erectile dysfunction	Right ear/Clark IV	T1WI high signal, T2WI low signal, heterogeneous enhancement	Surgery + radiotherapy	60	Survived 7 months	NA
3	Jung et al. /2007 (18)	M/70	Visual impairment	Left great toe	T1WI isointense, T2WI hyperintense, homogeneous enhancement. Several small foci within the tumor mass showed T1WI high signal and T2WI low signal without enhancement, suggestive of subacute hematoma or melanin	Surgery	15	Died 1 month later	NA
4	McCutcheon et al. /2007 (19)	M/77	Ptosis and diplopia, visual impairment	Anterior chest/Clark IV	T2WI isointense	Surgery + radiotherapy	33	Survived 6 months	NA
5	McCutcheon et al. /2007 (19)	M/42	Diabetes insipidus, visual impairment	Anterior chest/Clark IV	T2WI signal isointense with slight hypointensity	Surgery + radiotherapy	77	Died 4 months later	NA
6	Guzel et al. /2009 (20)	F/46	Headache	Left shoulder	T1WI isointense, T2WI isointense, enhancement	Biopsy + chemotherapy	84	Died 12 months later	NA
7	Kano et al. /2009 (21)	M/47	Diabetes insipidus	NA	NA	Surgery + radiotherapy	NA	Died 34.8 months later	NA
8	Kano et al. /2009 (21)	F/52	Oculomotor palsy	NA	NA	Surgery + radiotherapy	NA	Died 21.8 months later	NA
9	Masui et al. /2013 (7)	M/68	Headache and visual impairment, pituitary apoplexy	Stomach	T1WI high signal, T2WI low signal, heterogeneous enhancement	Surgery	Simultaneously	Lost to follow-up 2 months later	NA
10	Zoli et al. /2013 (22)	NA	Visual impairment	NA	NA	Surgery + radiotherapy	NA	NA	NA
11	Burkhardt et al. /2016 (23)	M/73	Visual impairment, anterior pituitary dysfunction, diabetes insipidus	NA	NA	Surgery + radiotherapy	NA	Died 8 months later	NA
12	Yang et al. /2017 (24)	F/62	Visual impairment	Left heel	T1WI isointense, T2 WI isointense, homogeneous enhancement	Surgery + hormone replacement	24	Survived 22 months, in poor health	NA
13	Ramos et al. /2017 (28)	M/67	Headache, vomiting, decreased left-eye vision	Back/Clark IV	Heterogeneous marked enhancement	Surgery	36	Died 3 months later	NA
14	Castle-Kirszbaum et al. /2018 (25)	M/78	Visual impairment, pituitary dysfunction	NA	T1WI high signal, T2WI low signal	Surgery	Simultaneously	NA	NA
15	Mattogno et al. /2020 (13)	M/32	Headache	Back	T1WI isointense with high signal spots, slightly high T2WI signal, heterogeneous enhancement	Surgery + targeted chemotherapy	120	No disease recurrence for 18 months	IHC positive
16	Mattogno et al. /2020 (13)	M/32	Diplopia, visual impairment with partial ptosis of the left eye	Right breast	T1WI high signal; T2WI low signal; heterogeneous enhancement	Surgery + interferon	84	Died 14 months later	IHC negative
17	Lithgow et al. /2020 (26)	F/64	Hypopituitarism, visual impairment	NA	NA	Surgery	84	Recurrence 45 months later	NA
18	Lithgow et al. /2020 (26)	F/56	Visual impairment	NA	NA	Monitoring	84	NA	NA
19	S. Ng et al. /2020 (14)	F/51	Visual impairment	NA	T1WI heterogeneous high signal; T2WI low signal intensity, with mild homogeneous enhancement	Surgery + BRAFi–MEKi	84	Died 12 months later	IHC positive
20	Mormando et al. /2020 (5)	M/33	Headache	Scapula/Clark IV	T1WI low signal, T2WI high signal	Surgery + BRAFi–MEKi	127	Recurrence 3 months after surgery, complete remission after BRAFi–MEKi treatment	Positive
21	Giuffrida et al. /2021 (12)	M/77	Loss of consciousness, visual impairment	Shoulder	T1WI isointense signal	Hormone replacement, pembrolizumab	24	Good prognosis at 21 months	NA
22	Lamorie-Foote et al. /2021 (15)	M/64	NA	Diffuse acral lentiginous melanoma	Heterogeneous marked enhancement, suggestive of hemorrhage	Surgery	24	Tumor progressed 3 months after surgery, died due to an accident	NA
23	Yang et al. /2023 (27)	M/72	NA	NA	T1WI signal low, T2WI high signal	Radiotherapy	16	Survived 42.7 months	NA
24	Present case	F/65	Dizziness	Lower leg	T1WI isointense to slightly high signal, slightly high T2WI signal. Progressive heterogeneous marked enhancement	Surgery + chemotherapy	120	Died 12 months later	Positive

M, male; F, female; MRI, magnetic resonance imaging; NA, not available; T1WI, T1-weighted imaging; T2WI, T2-weighted imaging; BRAFi, *BRAF* inhibitor; MEKi, MEK inhibitor; IHC, immunohistochemistry.

## Discussion

This study investigated the clinical, imaging, and pathological features of MM with pituitary metastasis and highlighted the diagnostic challenges associated with this rare condition. Through a comprehensive case analysis and literature review, we demonstrate the significant value of a multimodal diagnostic approach involving the integration of clinical, radiological, pathological, and molecular data in enhancing diagnostic accuracy and emphasize the need for a detailed patient history and a comprehensive diagnostic strategy.

MM is considered one of the most centrophilic tumors, with central nervous system metastases occurring in 10%–40% of patients with MM (30, 31). However, pituitary involvement remains rare. The literature reports several risk factors for the development of brain metastases in MM, including the primary tumor thickness (Breslow depth > 3 mm), the presence of ulceration, and the location of the primary tumor (32). In our case series, the primary site of MM was most commonly the skin (13 out of 14 cases), with the condition in all cases presenting as Clark stage IV cancer, with a Breslow depth ranging from 1.5 to 12 mm. The median time to MM brain metastasis is reported in the literature as 30 months (32). Of the 24 MM cases, the primary lesion was identified simultaneously in two cases (7, 25), and 15 cases had clearly documented metastasis to the pituitary. The median time from MM diagnosis to pituitary metastasis was 36 months.

The molecular mechanisms underlying brain metastasis in MM are multifaceted, encompassing oncogenic mutations, aberrant activation of signaling pathways, alterations in the intracranial microenvironment, and expression of nerve growth factor receptors (33, 34). We hypothesized that *BRAF* mutations may be associated with an increased propensity for pituitary metastasis in patients with MM. Studies have demonstrated that *BRAF* mutations activate downstream signaling via the MAPK pathway (35), which may indirectly regulate the expression of chemokine receptors such as CXCR4 and promote the migration of tumor cells to the CXCL12-rich pituitary microenvironment (36). *BRAF* is the most frequently mutated gene in melanocytic tumors, with approximately 50% of patients with metastatic MM harboring *BRAF* mutations, 95% of which are located in exon 15 at *BRAF* V600 (5). In our retrospective analysis, 80% (4 out of 5) of cases were *BRAF*-positive, indicating a potential association between MM with *BRAF* mutations and pituitary metastasis. However, this observation was based on only four mutation-positive cases and remains hypothesis-generating; larger studies are needed to establish any causal relationships.

The MRI features of MM with pituitary metastasis are dynamically influenced by melanin content owing to its paramagnetic properties. Melanin-rich tumors typically exhibit T1WI hyperintensity and T2WI hypointensity. In our case series, 38.9% (7 out of 18) of cases exhibited this typical “T1 hyperintensity/T2 hypointensity” pattern, while the present case displayed atypical slight T2 hyperintensity, likely related to a lower melanin content. These MRI features are closely associated with the pathological characteristics of MM, reflecting its growth and metabolic features within the pituitary gland. However, similar MRI features can also be observed in hemorrhagic pituitary macroadenomas (12, 37), making preoperative diagnosis challenging and necessitating close integration with clinical history.

In clinical practice, diagnosis is relatively straightforward when patients have a known history of MM. However, if the patient’s history of MM is unknown, the diagnosis becomes challenging due to the diverse histological structures and cellular morphologies of MM, which can readily be confused with those of other primary tumors. This diagnostic difficulty is further compounded in rare cases where the MM has metastasized to primary pituitary tumors, making differentiation particularly challenging. In the present case, the unknown MM history, along with significant microscopic morphological and immunophenotypic overlap with pituitary adenomas, contributed to the diagnostic difficulty.

Microscopically, the tumors exhibited typical morphologies, with pigment deposition observed in most cases. Among four cases involving collision tumors, three involved MM metastasis to a pituitary adenoma (15, 24, 28), and one involved metastasis to a pituitary eosinophilic tumor (18). Immunohistochemistry revealed diffuse positivity for vimentin, S-100, Melan-A, HMB45, and SOX10. *BRAF* V600E mutation detection also provided critical diagnostic support, If *BRAF* V600E immunohistochemistry is negative, PCR or NGS can be performed to confirm the mutation status. Of the 24 cases, 5 underwent *BRAF* testing (three via immunohistochemistry), and 4 exhibited positive results. There is significant overlap in the histological features of granulosa cell tumors, pituitary cell tumors, and spindle cell eosinophilic tumors. According to the 2021 World Health Organization (WHO) Classification of Tumors of the Central Nervous System (38), these tumors are grouped together due to their diverse cellular arrangements and morphologies, including epithelioid, round, and spindle-shaped cells with eosinophilic or pale pink cytoplasm, inconspicuous nucleoli, abundant interstitial blood vessels, and lymphocytic infiltration. Immunomarkers such as TTF-1, vimentin, S-100, and GFAP are highly specific to these tumors. While typically benign (WHO grade 1), atypia and mitosis can occur in recurrent cases (39), further increasing the difficulty of differential diagnosis. Therefore, integrating clinical history with immunohistochemistry and confirmatory molecular analysis (*BRAF* V600E) can be used to reliably distinguish metastatic melanoma from primary pituitary tumors.

Most brain metastases in MM are advanced at presentation, characterized by refractory disease and a poor prognosis (3). Follow-up data were available for 20 patients, with the follow-up durations ranging from 1 to 45 months. Among these patients, 11 died during the follow-up period, including 1 due to an unrelated accident. Of the 10 patients who died from disease-related causes, the survival times ranged from 1 to 34.8 months, with a median survival of 12 months. Among the remaining survivors, one patient survived for 22 months but with poor health. For brain metastases in MM, multimodal treatment approaches are recommended, including surgery, radiotherapy, hormone replacement therapy, chemotherapy (40), immunotherapy, and targeted drug therapy (12, 15). *BRAF* mutation testing not only aids in diagnosing MM but also provides a theoretical basis for targeted therapies. Small-molecule inhibitors targeting the *BRAF* V600 mutation have been shown to have remarkable efficacy (41), and the combination of a *BRAF* inhibitor (*BRAF*i) with a MEK inhibitor (MEKi) can mitigate drug resistance and improve prognosis. Among the cases retrospectively analyzed in this study, patients who received *BRAF*i–MEKi combination therapy achieved remission (5). Currently, three *BRAF*i–MEKi combination regimens—dabrafenib with trametinib, encorafenib with binimetinib, and vemurafenib with cobimetinib—are considered the standard treatment for advanced *BRAF*-mutated MM.

This study has some limitations. First, some of the case reports lacked detailed clinical follow-up information, and the literature review may be subject to publication bias, which limits the generalizability of our findings. Second, molecular data were incomplete for some patients, which may restrict the depth and universality of our analysis. Future studies should address these limitations by incorporating more comprehensive clinical and molecular data.

## Conclusion

Our findings underscore the rarity of pituitary metastasis in MM and the complexity of its diagnosis. Despite numerous challenges, meticulous clinical observation, imaging, and pathological examination led to accurate diagnosis. A thorough patient history and comprehensive approach are essential when facing atypical presentations, and further studies are needed to identify early diagnostic markers.

## Data Availability

The original contributions presented in the study are included in the article/supplementary material. Further inquiries can be directed to the corresponding author.
